# Efficacy of silver diamine fluoride (SDF) in arresting dentin caries against inter-kingdom biofilms of *Streptococcus mutans* and *Candida albicans*

**DOI:** 10.1371/journal.pone.0308656

**Published:** 2024-09-30

**Authors:** Suphanida Kaewkamchai, Panida Thanyasrisung, Waleerat Sukarawan, Lakshman Samaranayake, Nozimjon Tuygunov, Siriporn Songsiripradubboon

**Affiliations:** 1 Department of Pediatric Dentistry, Faculty of Dentistry, Chulalongkorn University, Bangkok, Thailand; 2 Department of Microbiology and Center of Excellence on Oral Microbiology and Immunology, Faculty of Dentistry, Chulalongkorn University, Bangkok, Thailand; 3 Faculty of Dentistry, University of Hong Kong, Sai Ying Pun, Hong Kong; 4 Faculty of Dentistry, Chulalongkorn University, Bangkok, Thailand; 5 Faculty of Dentistry, University of Hong Kong, Hong Kong; University of Pennsylvania, UNITED STATES OF AMERICA

## Abstract

**Objectives:**

To compare, *in vitro*, the efficacy of three proprietary silver diamine fluoride (SDF) products in mitigating progression of dentinal caries induced by an inter-kingdom, dual-species, bacterial-yeast biofilm.

**Methods:**

Human dentin blocks were demineralized to create artificial caries lesions and randomized into three SDF test groups: Saforide, Topamine, T-SDF, and an aqueous control (n = 26 per group). After application of foregoing SDF variants, the blocks were incubated with *Streptococcus mutans* and *Candida albicans* for 24 h for biofilm development, and subsequently subjected to a microbe-induced, pH-cycling process for 7 days, to mimic the oral eco-system. The biofilm cell viability and surface topography were assessed by colony-forming units (CFUs) and scanning electron microscopy respectively. The lesion depth and mineral density were evaluated by micro-computer tomography. SDF precipitate and matrix-to-mineral ratio were evaluated by X-ray diffraction and Fourier transform infrared spectroscopy, respectively. Standard, accepted methodology was used for all these evaluations and procedures.

**Results:**

After pH cycling, the SDF groups demonstrated comparable inhibition of the biofilm relative to the control. the log CFU of *S*. *mutans* for Saforide, Topamine, T-SDF, and control were 6.69±0.73, 6.48±0.56, 6.63±0.66, and 8.01±0.45, respectively. For *C*. *albicans*, the log CFU were 4.86±0.44, 4.72±0.53, 4.92±0.29, and 5.60±0.27, respectively. The log CFU of *S*. *mutans* and *C*. *albicans* in the SDF groups were significantly lower than the control group (*p*<0.001). Further, the lesion depth decreased by approximately 14.79±7.00% in the SDF groups, while it increased by 11.07±8.61% in the control (*p*<0.001), and the mineral density increased by 16.36±4.58% in the SDF group, as opposed to a 5.59±2.64% reduction in the control (*p*<0.001) implying their caries mitigating effect. These findings were corroborated by SEM images of the lesions.

**Conclusion:**

SDF significantly mitigated dentin caries due to an assault by a polymicrobial plaque biofilm whilst arresting mineral loss and lesion growth. There was no difference in the caries-arresting efficacy of the compared SDF variants.

## Introduction

Dental caries, particularly in childhood, is a major global oral health problem in preschool children [1]. Neglecting treatment of caries as well as early childhood caries (ECC) can adversely impact the quality of life, overall health, development, and growth of children [2, 3].

Caries is a multifactorial disease, with dental plaque widely recognized as a prominent risk factor [4]. Mutans streptococci play a pivotal role in the development of dental caries as they are acidogenic and aciduric. In addition, they produce copious amounts of polysaccharides or glucans in the presence of fermentable carbohydrates, which facilitates bacterial adhesion to the tooth surfaces and contributes to the development of the EPS matrix of the plaque biofilm [5]. Recent findings also indicate that apart from bacteria, fungi belonging to the *Candida* species are associated with ECC and severe early childhood caries (S-ECC) as these eukaryotes possess many cariogenic attributes, including acidogenicity and aciduricity [6]. For instance, plaque biofilm of children with ECC have more significant quantities of *Candida* species compared to those with fewer or no dental caries [7, 8]. The inter-kingdom interactions between bacteria and fungi appear to promote cariogenicity. Sampaio et al. found in an *in vitro* study that dentin specimens exposed to acid with both *Streptococcus mutans* (*S*. *mutans*) and *Candida albicans* (*C*. *albicans)* exhibited significantly higher mineral loss compared to samples exposed to acid challenges with only the streptococci [9]. Previous study has also shown that *C*. *albicans* prevalence and abundance in ECC is directly proportional to the incidence and severity of ECC [10].

One major issue confronting dental professionals in the clinical management of childhood caries is the reluctance of the children and even their parents to visit the dentist due to dental anxiety and incorrect perceptions of the value of the deciduous dentition. In addition, patient access to healthcare services and treatment costs contribute significantly to delays in receiving treatment for dental caries in most of the affected jurisdictions [11, 12]. For caries management, non-invasive or minimally invasive techniques are considered cost-effective. These entail controlling lesion progression, promoting remineralization, and performing restorations only when necessary [13]. Topical application of silver diamine fluoride (SDF) is such a non-invasive dental treatment currently gaining popularity worldwide. SDF can arrest the progression of dentin caries and promoting significant remineralization, in both the primary as well as the permanent dentition [14, 15]. Furthermore, SDF can be applied topically without removing preexisting carious tissue and extensive instrumentation, thus encouraging treatment compliance in children [16].

A number of SDF products are currently available in the market with varying concentrations of the active ingredient, ranging from 12% to 38%. It is known that 38% of SDF has the highest efficacy in arresting carious lesions [17]. The anti-caries mechanism of SDF is now known to be multipronged due to its fluoride content and the constituent elemental silver ions. Briefly, the fluoride ions promote remineralization while inhibiting demineralization and, in addition reduces the acid tolerance of bacteria [6]. Silver ions are also antimicrobial in nature as they disrupt bacterial cell walls, leading to enzyme denaturation and inhibition of bacterial DNA replication [18]. Further, silver also inhibits biofilm formation by interfering with the glucosyltransferase enzyme pathway, which converts sucrose to glucans, a significant component of the biofilm’s extracellular polysaccharide (EPS) shell [19, 20].

However, the effect of SDF on the progression of dentin carious lesions in the presence of dual species of bacterial-yeast biofilm is not known. There is indeed, to our knowledge, no data on the efficacy of SDF in arresting dentine caries using in vitro models. Hence, in this study, we evaluated, for the first time, the efficacy of three SDF products in mitigating the progression of artificially created dentinal caries lesions by an inter-kingdom, polymicrobial biofilm of *S*. *mutans* and *C*. *albicans*. A modification of a previously described artificial pH-cycling experimental methodology described by Yu et al. was used for dentin caries arrest under a dual-species microbial pH-cycling challenge [21]. The opportunity was also taken to compare the anti-caries activity of three 38% of SDF variants available in Thailand.

## Materials and methods

### Sample preparation and artificial caries formation

**Fig 1:**
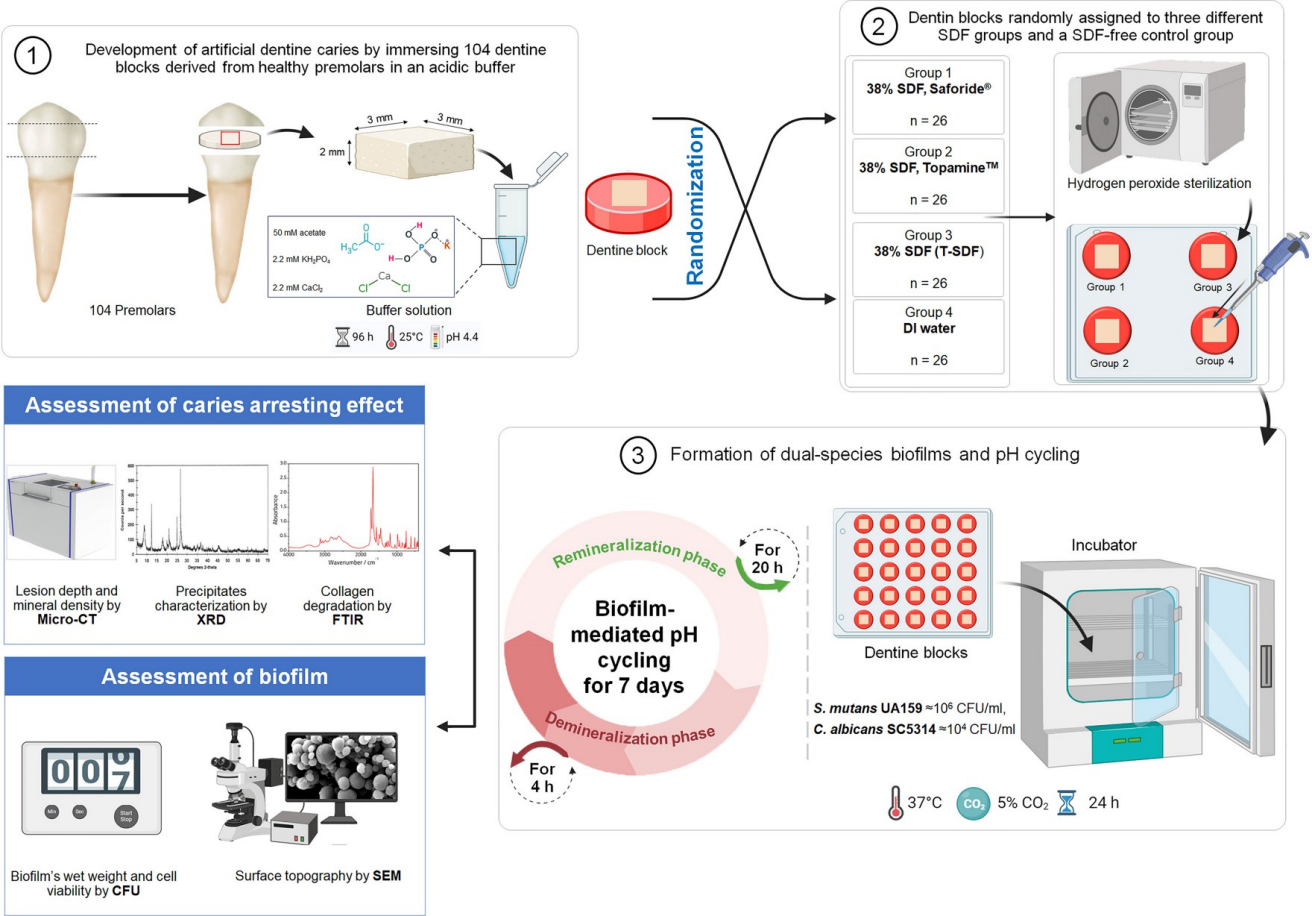
The schematic illustration of the experimental process. Reprinted from Created with BioRender.com, under a CC BY license, with permission from BioRender, original copyright 2024.

A total of 104 sound human premolars extracted for orthodontic reasons were collected in January 2022 from the dental department of Chum Phuang Hospital in Nakhon Ratchasima, Thailand. Patients provided verbal consent, understanding their teeth might be used for dental study or research, then documented in the patients’ records. Written informed consent was obtained from an authorized representative. The study protocol was approved by the Ethics Committee of the Faculty of Dentistry, Chulalongkorn University (HREC-DCU 2021–095) and Institutional Biosafety Committee (DENT CU-IBC 026/2022). The teeth were stored in 0.9% normal saline until used [22]. Each tooth was prepared into 3 x 3 x 2 mm^3^ dentin blocks. The surfaces of the blocks were polished using micro-fine 4000 grid silicon carbide abrasive paper and covered with acid-resistant nail varnish, except for the experimental window of 2 x 2 mm^2^. (Fig 1).

The sample size was determined based on the previous study by Mei et al. [23] using G*Power software (version 3.1.9.4). The calculation was based on an omnibus F test with an adjusted effect size of 0.5, an alpha value of 0.05, and a power of 0.80. As a result, a total of 12 samples per group were required. To account for laboratory errors, this study used 14 samples per group for the caries arresting effect test and 12 samples per group for the biofilm assessment.

The specimens were immersed in an acetate buffer solution containing 2.2 mM CaCl_2_, 2.2 mM KH_2_PO_4_ and 50 mM acetate at pH 4.4 for 96 h at 25°C to create artificial carious lesions (220–240 μm in depth) as described by Mei et al. [23]. The specimens were then measured for baseline lesion depth (LD) and baseline mineral density (MD) by using micro-computed tomography (micro-CT). All specimens were sterilized with hydrogen peroxide plasma (STERRAD, 100NX^TM^ Sterilization System). The flow chart in Fig 1 summarizes the protocol of the study. (The overall procedure and number of samples in each assessment were provided in S1 Fig).

### Interventions

The dentin blocks were randomly allocated into four experimental groups of 26 blocks each. The blocks in Groups 1 to 3 received topical applications of 38% SDF, Saforide (Toyo Seiyaku Kasei Co., Ltd., Osaka, Japan), 38% SDF, Topamine (DentaLife, Thailand) and a locally produced 38% SDF (T-SDF), respectively. All 38% SDF were in clear liquid form except for Topamine, which was a blue solution, with a pH range of 9.0–10.0. The Group 4 blocks were the SDF-free controls, suspended in sterile deionized water.

To mimic the clinical scenario, a 5 μl of the SDF solution was applied onto the experimental “caries window” area by using an automatic pipette, and a micro brush was then used to gently rub the solution over the caries lesion for a period of one minute. The solution was left on the dentin surface for two minutes before the excess was rinsed thoroughly with deionized water in a standardized manner. The control blocks were similarly treated with deionized water.

### Organisms, dual-species biofilm development and pH-cycling

*S*. *mutans* UA159 and *C*. *albicans* SC5314 were obtained from the archival collection of the Department of Microbiology, Faculty of Dentistry, Chulalongkorn University. The identity of the isolates was confirmed before their use. The 16 h overnight culture of *S*. *mutans* UA159 and *C*. *albicans* SC5314 were inoculated in Brain Heart Infusion (BHI) broth and yeast extract peptone dextrose (YPD) broth, respectively. The cultures were incubated at 37°C until they reached the mid-log phase growth (OD_600 nm_ ≈ 0.5), the cell was then harvested and resuspended in BHI containing 1% of sucrose to a concentration of 10^6^ CFU/ml for *S*. *mutans* and 10^4^ CFU/ml for *C*. *albicans* [9]. This ratio of organisms is comparable to that in saliva samples in ECC [24, 25].

To mimic the salivary pellicle, the specimens were mounted on the lid of 24-well plate and immersed in artificial saliva prepared for one hour at 37°C as described by Silva et al. [26]. Then, each dentin block was inserted into a single well of a flat-bottomed 24-well plate containing the microbial inoculum (2 ml per well) and further incubated at 37°C for 24 h in a milieu of 5% CO_2_ to obtain a mature biofilm.

The pH-cycling adopted in this study was a modification of a method described by Yu et al. [21]. The dentin specimens went through a 4 h demineralization period and a 20 h remineralization period for 7 days. To simulate the demineralization process, each dentin specimen was incubated in 2 ml of BHI containing 1% of sucrose for 4 h at 37°C with 5% CO_2_. Afterward, the suspending medium was collected, and the pH determined. The specimens were rinsed with sterile phosphate buffer saline solution (PBS) and were then incubated with 2 ml of remineralization solution (prepared by 0.5 mmol/L CaCl_2_, 0.9 mmol/L K_2_HPO_4_, 130 mmol/L KCl, and 20 mmol/L HEPES, and then adjusted to pH 7.0 with 1 mmol/L KOH) at 37°C with 5% CO_2_ for 20 h [21]. This process was sequentially repeated on seven occasions, on 7 consecutive days.

### Assessment of caries arresting effects

#### Lesion depth (LD) and mineral density (MD)

After pH-cycling, the specimens in each group were scanned using micro-CT (μCT35; Scanco Medical AG, Brüttisellen, Switzerland) to measure the LD and MD [22]. The X-ray source operated at a source voltage of 70 kV and a current of 114 μA and was assessed at medium resolution (1024 x 1024). To mitigate beam hardening effects, a 0.5 mm-thick aluminum filter was positioned in the beam path [22].

To calibrate the micro-CT, a series of hydroxyapatite phantom standards with a range of densities were scanned. Cross-sectional images of each dentin specimen were obtained from reconstructed images. From these cross-sectional images, 10 were randomly selected to evaluate the lesion depth. Image analysis was conducted using the software Image J (National Institutes of Health, MD, USA). An image area with a grayscale value of more than 95% was defined as sound dentin [27]. A standardized single examiner determined an area of demineralized dentin, and the depths of the lesions were measured.

The MD of each specimen (mgHA/cm^3^) was determined by volumetric measurements. Two-dimensional cross-sectional images of the specimens were acquired, and these images were then reconstructed into a three-dimensional model using the Micro-CT evaluation program. The volumes of interest (VOIs) with dimensions of 150 x 150 x 300 voxels were defined by selecting a section of the carious lesion that was not at the edges of the lesion, for the final evaluation [28].

#### X-ray diffraction analysis of precipitate

Two blocks from each group were used to study the characteristics of the precipitates derived from XRD analyses. The data were collected with an X-ray powder diffractometer (D8 DISCOVER; Bruker AXS, Karlsruhe, Germany) equipped with a Cu-K_α_ (I = 1.5418 A˚) radiation detector. The X-ray generator operated at an accelerating voltage of 40 kV and a current of 40 mA, respectively. The data was collected with 2θ ranges from 20° to 60° [23].

#### Matrix to mineral ratio

Six blocks from each group were used to analyze variations in the chemical structure, including the organic content of the dentin caries lesions. For this purpose, a Fourier transform infrared (FTIR) spectroscopy (FTIR Spectrum-One; PERKIN ELMER, Burladinge, Germany) equipped with an attenuated total reflection element was used. The wavelength number of the infrared radiation ranged from 650 cm^-1^ to 4000 cm^-1^. The extent of demineralization of the dentin caries lesions was studied by evaluating the ratio of the integrated area of collagen amide I absorbance (between 1585 and 1720 cm^-1^) to that of HPO_4_^2-^ absorbance (between 900 and 1200 cm^-1^) [29].

### Quantitative characteristics of the biofilm

#### Biofilm cell viability and wet weight

After 7 days of pH-cycling, the biofilm growth on the samples were harvested in a standard manner by a single examiner, by thorough scraping with a sterile pipette tip, and vortexed in 1 ml of PBS buffer. An aliquot of 100 μl of the sonicated biofilm suspensions was serially diluted 10-fold and spread on Mitis Salivarius agar plate and Yeast Peptone Dextrose agar plates to determine the CFU of *S*. *mutans* and *C*. *albicans*, respectively. These plates were incubated at 37°C with 5% CO_2_ for 24 h before the quantification of the CFUs [29].

To derive the biofilm wet weight, an aliquot of 500 μl of the sonicated biofilm suspensions was transferred to previously weighed tubes. Subsequently, this sample was centrifuged at 10,000 g for 5 min at 4°C. The supernatant was carefully removed, the pellet was weighed, and the biofilm wet weight was determined after volume normalization [9].

#### Lesion topography

Dentin blocks with and without the biofilm were fixed in 2.5% glutaraldehyde solution at 4°C for 24 h, then rinsed with PBS. They were serially dehydrated with ethanol solutions, dried in a desiccator and sputter coated with gold. The topographical features of the surface were observed under scanning electron microscopy (SEM) (Quanta 250; FEI Company, Eindhoven, The Netherlands).

### Statistical analysis

All the data were assessed for a normal distribution using the Shapiro-Wilk test for normality. To compare the data at baseline and after cycling, the Wilcoxon signed-rank test was applied for lesion depth, and a two-tailed paired t-test was used for mineral density, based on the data distribution. One-way ANOVA followed by the Games-Howell test and Bonferroni post-hoc test was used to compare mineral density and lesion depth among the four treatment groups. The ratio of amide I: HPO_4_^2−^ absorbance, log_10_ CFU, and biofilm’s wet weight were compared using the Kruskal-Wallis test, followed by Dunn’s test. All statistical analyses were conducted using IBM SPSS Version 23.0 software (IBM Corporation, Armonk, New York, USA), with a significance level set at 5%.

## Results

### pH-cycling data

After a 4 h demineralization period, the pH of the suspending medium in the three SDF-treated groups decreased on average from 7.36 to 6.35 (±0.21) over 7 cycles. While the SDF-free control group had a lower pH value, with a mean pH of 6.21 (±0.11).

After a 20 h remineralization period, the pH of the media in all groups was higher than the critical pH of dentin demineralization mimicking the *in vivo* environment. The mean pH of the three SDF-treated groups and the SDF-free control group was 6.80 (±0.04), and 6.80 (±0.08), respectively.

### Assessment of dentin demineralization

#### Lesion depth (LD) and mineral density (MD)

**Fig 2:**
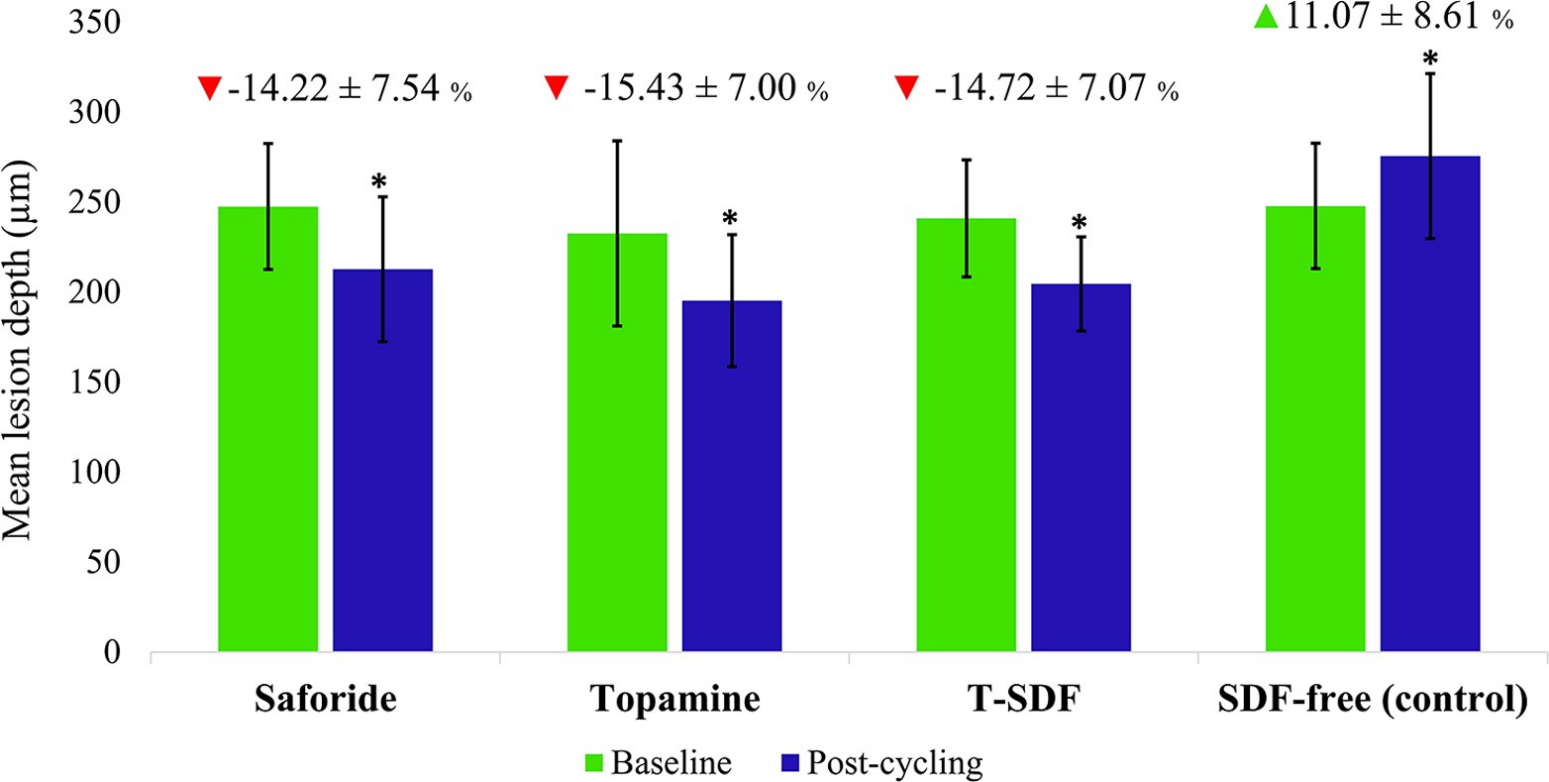
Mean lesion depth of the SDF test groups and the SDF-free group at baseline and post-cycling time points. * Differences in mean lesion depth between baseline and post cycling, *p*<0.05, (Wilcoxon Signed ranks test). The percentage change of lesion depth is indicated by triangles (mean ± SD). Red and green triangles indicate significant differences among the test groups and the control, *p*<0.001 (One-way ANOVA and Bonferroni post-hoc correction).

After pH-cycling, significant changes were observed in the mean LD values of all groups. Specifically, the three experimental groups Saforide, Topamine, and T-SDF exhibited a decrease in LD, whereas the SDF-free control group showed an increase in LD compared to their respective baselines. Saforide, Topamine, and T-SDF showed decreased of 14.22±7.54% (95% CI, 9.87 to 18.58), 15.43±7.00% (95% CI, 11.39 to 19.48), and 14.72±7.07% (95% CI, 10.64 to 18.80), respectively, while the control increased by 11.07±8.61% (95% CI, 6.09 to 16.04). The three SDF groups showed significant percentage changes in LD in comparison to the SDF-free control (*p*<0.001; Fig 2). The mean difference between Saforide and the SDF-free control was -25.29 (95% CI, -33.15 to -17.43). The mean difference between Topamine and the SDF-free control was -26.50 (95% CI, -34.36 to -18.64). The mean difference between T-SDF and the SDF-free control was -25.79 (95% CI, -33.65 to -17.92). There was no significant difference in the percentage changes of LD between the three SDF groups.

**Fig 3:**
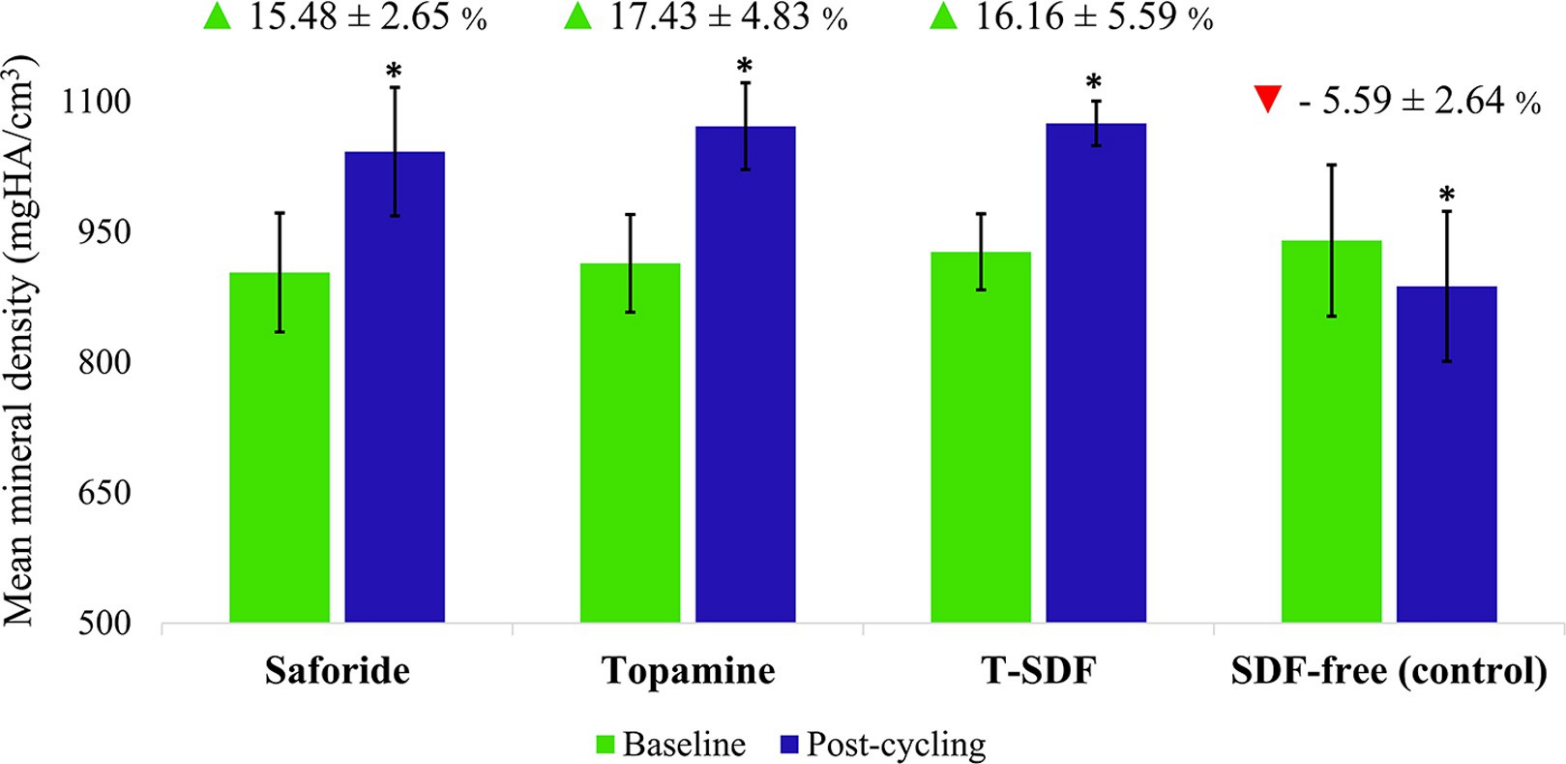
Mean mineral density of the SDF test groups and the SDF-free group at baseline and post-cycling time points. * Differences in mean mineral density between baseline and post cycling, *p*<0.05, (Paired *t*-test). The percentage change of mineral density (mean ± SD) is indicated by triangles. Red and green triangles indicate significant differences among the test groups and the control, *p*<0.001 (One-way ANOVA and Games-Howell post-hoc correction).

Saforide, Topamine, and T-SDF groups showed a statistically significant increase in MD, while the control group showed a significant decrease in MD after pH-cycling. There were significant changes in the percentage changes in MD of all three SDF groups in comparison to the control after the pH-cycling process (*p*<0.001; Fig 3). Thus, Saforide, Topamine, and T-SDF showed increased percentage changes in MD of 15.48±2.65% (95% CI, 13.95 to 17.01), 17.43±4.83% (95% CI, 14.64 to 20.22), and 16.16±5.59% (95% CI, 12.93 to 19.39), respectively, while the control decreased by 5.59±2.64% (95% CI, -7.12 to -4.06). The mean difference between Saforide and the SDF-free control was 21.06 (95% CI, 18.32 to 23.80). The mean difference between Topamine and the SDF-free control was 23.02 (95% CI, 18.90 to 27.13). The mean difference between T-SDF and the SDF-free control was 21.75 (95% CI, 17.09 to 26.40). There was no significant difference in the percentage changes in MD between the three SDF groups.

#### Precipitates

**Fig 4:**
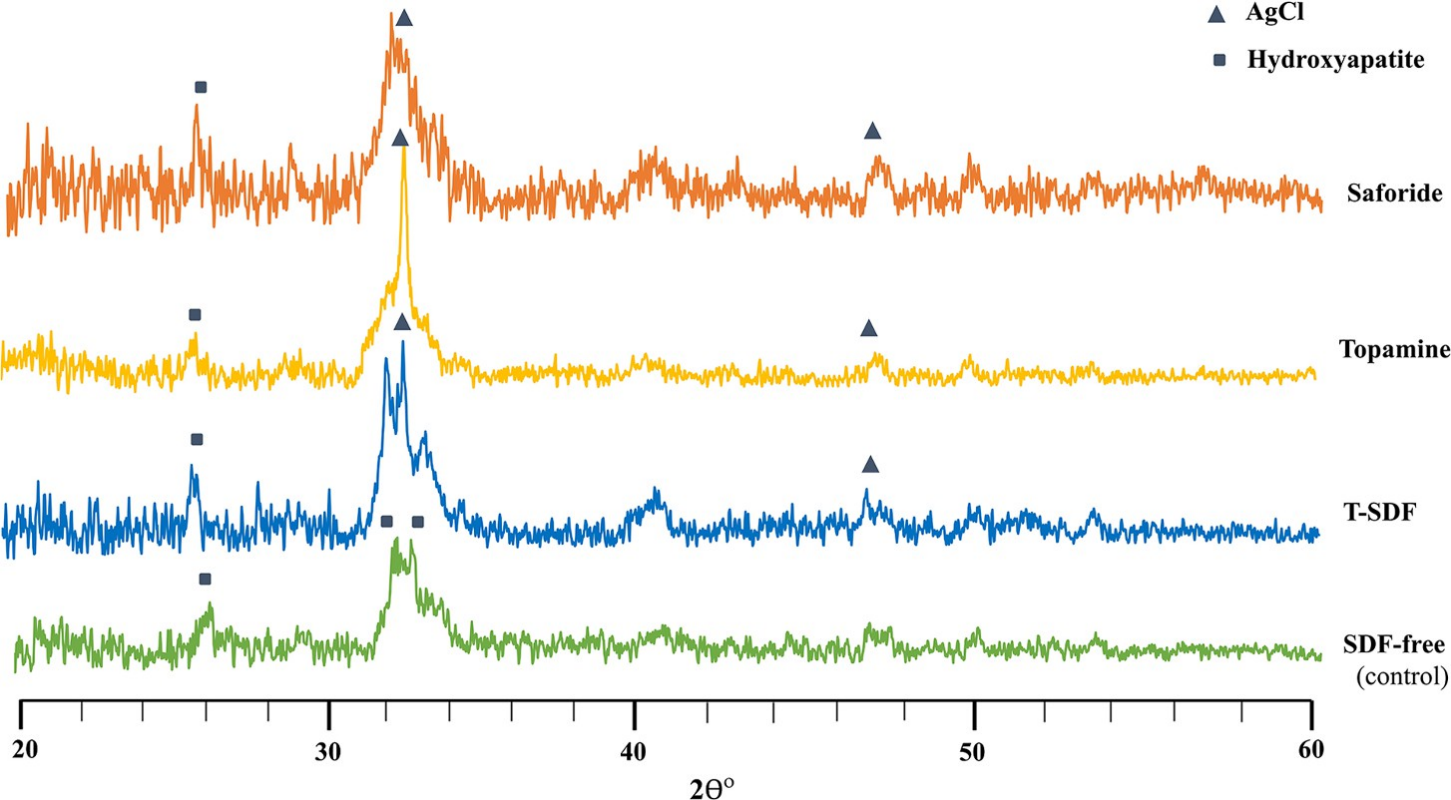
Typical X-ray diffraction patterns of the dentin of the three SDF treated groups and the control group. Note the reduced AgCl peak (triangle) of the control group compared with the SDF-treated test groups.

Evaluation of the X-ray diffractometry profiles of the four groups showed characteristic diffraction peaks corresponding to silver chloride (AgCl) at 32.2° and smaller peaks representing hydroxyapatite (HAP) at 46.3° (Fig 4). Apart from HAP, the three SDF-treated groups also demonstrated the presence of diffraction peaks in proximity to hydroxyapatite, corresponding to fluorapatite development. However, the control group displayed only the HAP peak.

#### Matrix to mineral ratio

The mean values of the ratio of amide I: HPO_4_^2−^ absorbance (±SD) of Saforide, Topamine, T-SDF, and the control group were 0.66 (± 0.21), 0.56 (± 0.11), 0.55 (± 0.10), and 0.72 (± 0.16), respectively. The ratio of amide I: HPO_4_^2−^ absorbance of the three SDF-treated groups was lower compared to the control group, though not to a significant extent. This implied more mineral precipitation and less collagen exposure at SDF-exposed sites.

### Biofilm characteristics

#### Biofilm cell viability and wet weight

There was no significance in the Log CFU of *S*. *mutans* and *C*. *albicans* counts, as well as the weight of dual-species biofilms for the three treatment groups after 7 days of pH-cycling (Table 1). However, the control group demonstrated significantly higher Log CFU for both *S*. *mutans* and *C*. *albicans* compared to all three SDF-treated groups (*p*<0.001). The biofilm weight of the control group was also significantly higher than the three SDF-treated groups. The SEM images corroborated the findings.

**Table 1:** The viability of *S*. *mutans* and *C*. *albicans* (log CFU/ml) and the wet weight of dual-species biofilms (mg) of the experimental and the control groups after the pH-cycling procedure.

Treatment groups (n = 10/group)	Biofilm cell viability (log CFU/ml)				Wet weight of biofilm (mg)	
	*S*. *mutans* UA159		*C*. *albicans* SC5314			
	mean ± SD	95% CI	mean ± SD	95% CI	mean ± SD	95% CI
**Saforide**	6.69 ± 0.73 ^a^	6.08–7.20	4.86 ± 0.44 ^c^	4.54–5.17	2.36 ± 0.82 ^e^	1.79–3.06
**Topamine**	6.48 ± 0.56 ^a^	6.02–6.87	4.72 ± 0.53 ^c^	4.34–5.10	2.55 ± 1.03 ^e^	1.69–3.08
**T-SDF**	6.63 ± 0.66 ^a^	6.16–7.18	4.92 ± 0.29 ^c^	4.71–5.13	3.24 ± 1.19 ^e^	2.64–4.24
**SDF-free (control)**	8.01 ± 0.45 ^b^	7.79–8.39	5.60 ± 0.27 ^d^	5.40–5.79	7.69 ± 3.00 ^f^	6.17–10.81

CI = confidence interval.

Within a column, the difference of superscript letters indicates the statistically significant difference (*p*<0.05, Kruskal-Wallis test with Dunn’s post-hoc correction).

#### Lesion topography

**Fig 5:**
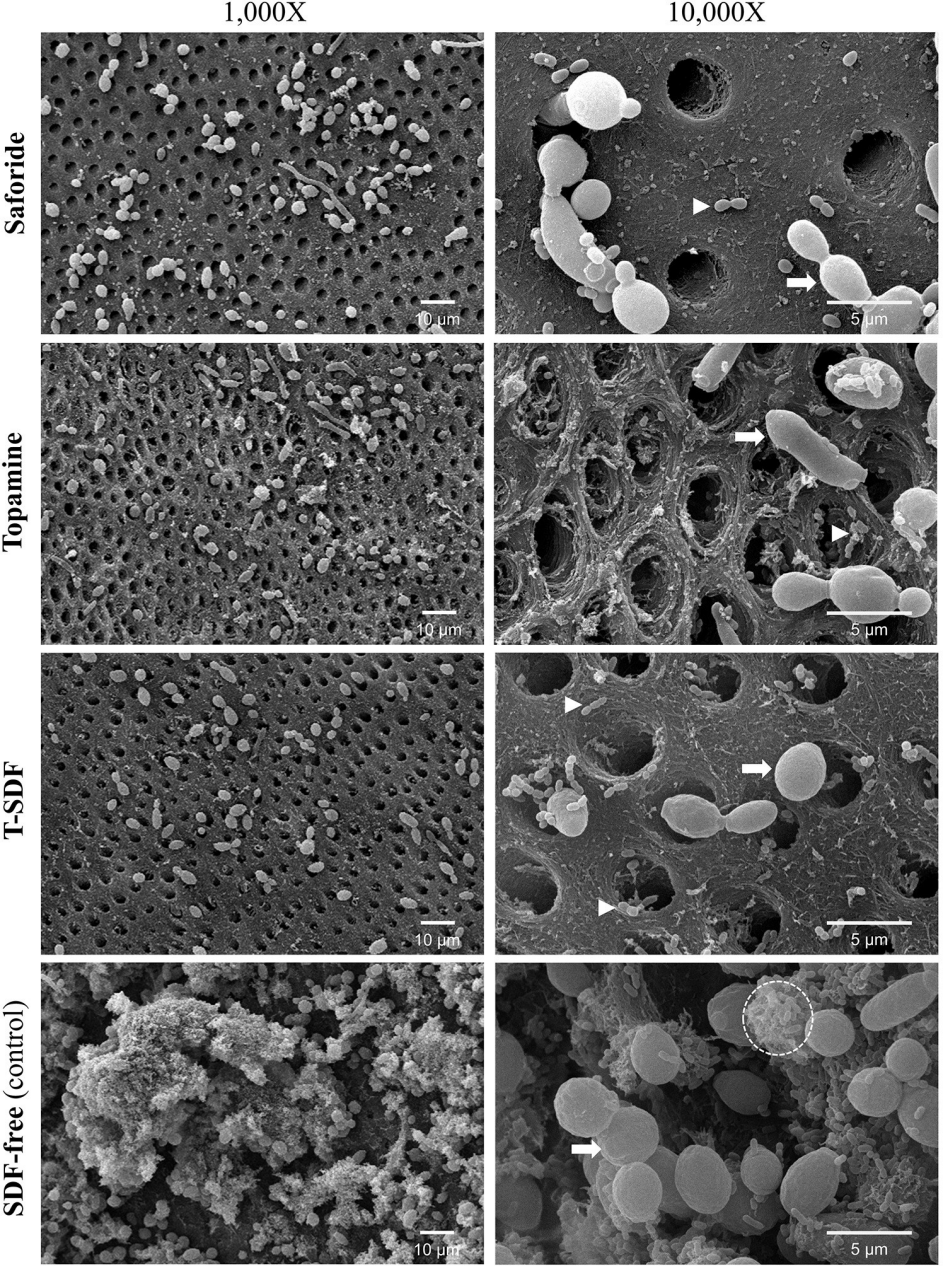
Dual-species biofilms of *S*. *mutans* and *C*. *albicans* on the dentin surface after pH cycling study showing sparse biofilm development on SDF exposed dentin and profuse interkingdom biofilms on the control SDF free control samples (bottom panel); white triangle: *Streptococcus mutans*; white arrow: *Candida albicans*; circle: Aggregates of streptococci.

**Fig 6:**
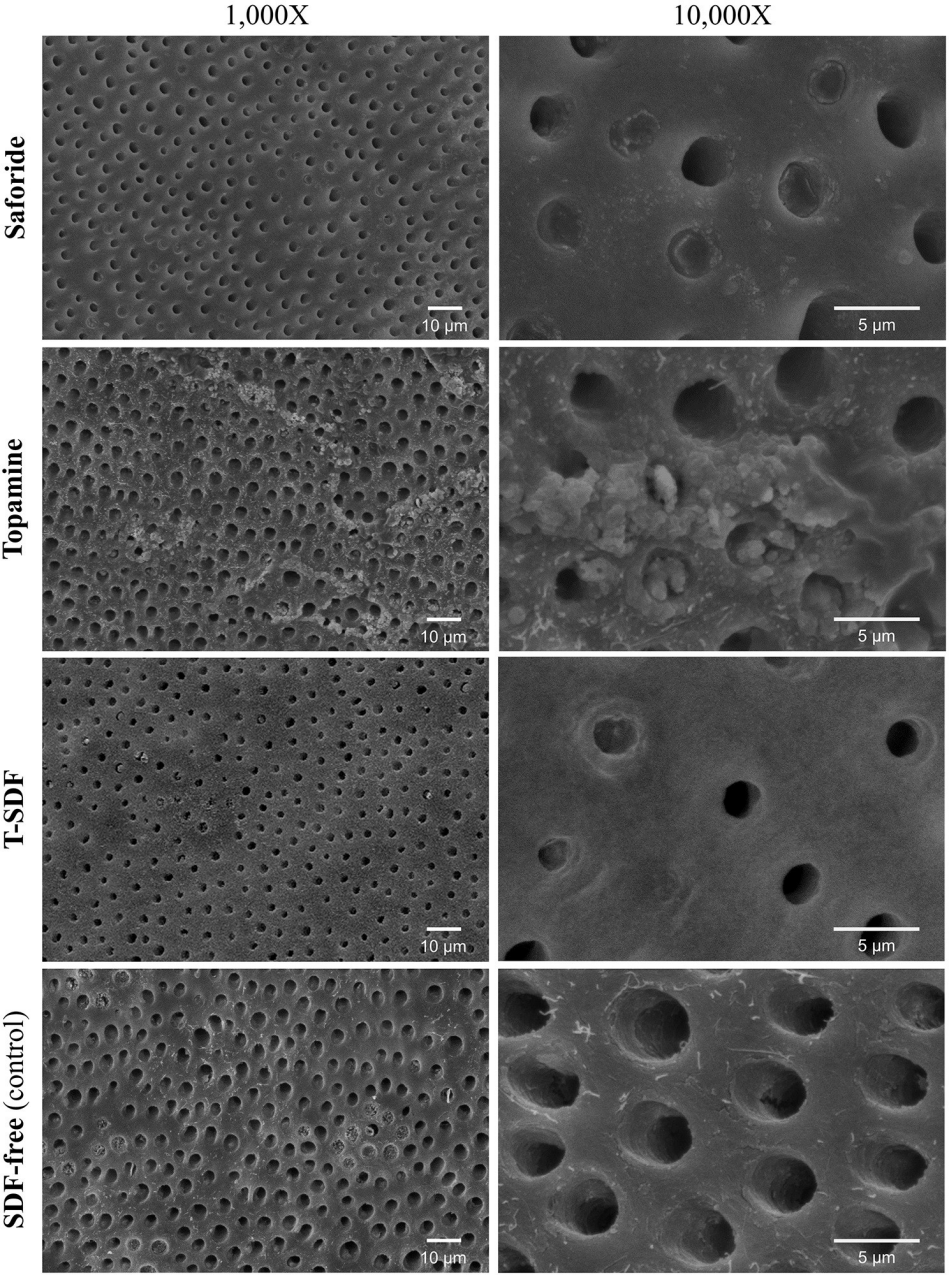
Representative SEM images of the dentin surface topography of the three SDF test groups and the SDF-free group. Appearance of the dentin surface after removing the biofilm, after 7 days of pH cycling experiments. The exposed dentinal tubule orifices are clearly visible.

We evaluated the dentin surfaces before and after the removal of the biofilm growth of each of the SDF groups and the control group (Figs 5 and 6) after pH cycling to visually assess the mineral and the organic (collagen) content. Fig 5 illustrates the representative biofilm morphology on the dentin surfaces of the four groups. The dentin surface of control groups exhibited complete dual-species biofilm cover, characterized by the confluent growth of *S*. *mutans* and *C*. *albicans* in contrast to those of the SDF treated surfaces that had patchy biofilm growth with sparse, organism-free regions. On further examination, Topamine and T-SDF groups showed relatively rough surfaces with some exposed collagen, in comparison to the Saforide group with relatively smooth dentinal surfaces. Fig 6 clearly demonstrates the characteristic morphology of dentin with the dentinal tubules. Dentin surfaces treated with Saforide and the T-SDF showed crystalline precipitates covering the surface and partially occluding the dentinal tubules. Topamine group, on the other hand, showed an amorphous precipitate covering the intertubular dentin and peritubular dentin.

## Discussion

We report here for the first time that silver diamine fluoride (SDF) helps mitigate dentin caries development induced by inter-kingdom, polymicrobial plaque biofilms of *S*. *mutans* and *C*. *albicans*. Our aim was to reproduce the *in vivo* ecosystem as it is well known that the multispecies plaque biofilms resemble the natural lesional environment more closely than the mono-species biofilms [19, 30]. For this purpose and to mimic the oral ecosystem further, we used artificial saliva to coat the dentin surfaces. We believe that future workers could utilize our model to develop polymicrobial biofilms to better simulate the oral microbiome.

Although *mutans*-streptococci are traditionally considered to be the prime movers of the carious process recent data clearly indicate that the oral commensal eukaryotes belonging to *Candida* species play a pivotal role in its pathogenic process, particularly ECC and S-ECC [31]. Moreover, the *Candida* species isolated from such lesions are known to be diverse [7] and possess profuse acidogenic and aciduric traits that makes them ideal candidate cariogens [32]. Apart from the foregoing data, related studies have shown that coculture biofilms of *C*. *albicans* and *S*. *mutans* accelerated dentin demineralization and produced more profuse biofilms than mono-species biofilms [9, 33], and *in vivo* studies have revealed that co-infection with *S*. *mutans* and *C*. *albicans* can cause more extensive caries in rats exposed to a cariogenic diet than the either species alone [34]. However, there are no studies evaluating the role of such dual-species biofilms, and the impact of SDF on mitigating the caries challenge.

The metabolism of sucrose by *S*. *mutans* leads to a reduction in the pH of the medium due to the production of short chain carboxylic acids predominated by lactic acid [33]. While this study did not directly measure lactic acid, the pH levels we observed could be considered as an indicative pH level representative of *in vivo* conditions. We have shown that the mean pH of the media after demineralization period dropped from 7.36 to 6.21 (± 0.11) and 6.35 (±0.21) in SDF-free and SDF-treated groups. These values are lower than the critical pH of dentin (ranging from pH 6.7 to pH 6.5) [34–36]. Notably, the pH of the media in SDF-treated group had a higher pH than in SDF-free group. This difference may be due to the fewer microbial numbers in SDF-treated group, possibly due to the antimicrobial activity of SDF (Table 1 and Fig 5). Additionally, this higher pH level might clarify why the reduced lesion depth and the increased mineral density in the SDF-treated group (Figs 2 and 3, respectively).

The mitigation of biofilm growth on exposure to all three SDF-treated groups was clearly shown by the significant reduction in CFU of both *S*. *mutans* and *C*. *albicans*, as well as the biofilm wet weight. This was further confirmed by ultrastructural studies, which indicated profuse interkingdom bacterial-yeast biofilms in the control groups in comparison to sparse microbial growth in all SDF-coated specimens (Fig 5). SDF, therefore, effectively inhibits microbial growth in biofilms, leading to a lesser degree of acid production on exposure to dietary carbohydrates. Our data corroborates with the recent work of Takahashi et al., who also reported that the SDF-treated dentin surfaces demonstrated a quantitative and qualitative reduction of *S*. *mutans* biofilm than the control after 20 h of cariogenic biofilms formation in a computer-controlled oral biofilm reactor [35]. Another study by Sorkhdini et al. also found that biofilm treated with SDF had fewer *S*. *mutans* compared with those treated with silver nitrate or water [36].

The mechanistic reasons for the reduced microbial adhesion on SDF exposure could be the silver ions interacting with hydroxyapatite, leading to the formation of a silver hydroxyapatite layer that is less conducive to microbial cell adhesion [37]. Furthermore, silver ions could disrupt the glucosyltransferase enzyme pathway, preventing the microbial synthesis of glucans from sucrose, a compound essential for microbial adhesion to tooth surfaces [38, 39]. In addition, elemental silver is known to inhibit the production of extracellular phospholipases of *Candida*, a central pathogenic attribute of this yeast [40]. Yeast phospholipases play a critical role in their surface adhesion, yeast-to-hyphal phase transition, as well as tissue penetration, which are all essential requisites for successful host colonization [41, 42]. Thus, the phospholipase mediated suppression of adhesion may be one reason for the sparse yeast colonization we noted in the SDF-treated groups; the results are in line with the findings of Alshahni et al., who also found that the SDF-treated surfaces had significantly fewer adherent yeasts compared to SDF-free control [41].

While silver demonstrates strong antibacterial efficacy, high concentrations of fluoride, also impede cariogenic bacteria in dental plaque by directly inhibiting cellular enzymes or by augmenting the proton permeability of cell membranes [43]. However, the primary action of fluoride remains its remineralizing effect [44].

Another clinically relevant finding of our study was the diminished lesion depth of incipient caries lesions and a concomitant surge in surface mineral density of dentin after SDF application and subsequent pH-cycling. On the contrary, the SDF-free control showed increased lesion depth and decreased mineral density, indicating lesion progression after the pH cycling. According to some, the SDF-induced increased mineral density of the base layer of dentin is a result of the precipitation of calcium fluoride, silver phosphate, and elemental silver ions [18, 45]. This insoluble protective layer effectively reduces the loss of calcium and phosphorus from the carious lesions, thus retarding lesion progression [45]. Furthermore, this layer is thought to act as a reservoir of fluoride and phosphate ions, facilitating remineralization [18, 46]. Another observation of ours was the presence of silver chloride (AgCl) compounds on all SDF-treated surfaces, but not silver phosphate. This may be due to the tendency of silver phosphate to dissolve in an alkaline environment. Mei et al. reported that silver phosphate has a tendency to dissociate into silver (and phosphate) ions, which combine with chloride ions to form silver chloride. Compared with silver phosphate, silver chloride is more stable with lower solubility and hence detectable with diffractometry [23, 47].

The major component of the organic dentin matrix is collagen, which is the structural framework that maintains its integrity [48]. During the natural caries development process, dentin collagen is exposed through demineralization. This leads to deproteination and dissolution of the exposed collagen fibers, thereby setting in a vicious cycle that leads to the further demineralization of exposed mineral dentin. Such alterations in demineralization and deproteination of dentin is indicated by change in the FTIR signal [49]. Our FTIR studies indicated a reduction in the ratio of amide I: HPO_4_^2-^ of the three SDF groups compared to the control, suggesting a greater degree of mineral precipitation and indicating another protective benefit of SDF.

Finally, in comparing the two commercially available SDF suspensions, Saforide and Topamine, and the new locally produced variant T-SDF, we noted that all three brands, each containing the same concentration of SDF at 38% w/v, were equally effective in preventing demineralization of dentin by the dual-species microbial pH cycling challenges. In clinical terms dental professionals should be in a position to choose any of these commercially available products to manage caries. However, T-SDF, being a new development, requires further product evaluation in preclinical and clinical settings.

The dual-species biofilm model that we have used could be further extended in future studies to evaluate the effect of polymicrobial biofilms on the development and progression of dentinal caries and to evaluate topically applied anti-caries agents such as SDF derivatives or equivalent anti-caries compounds.

## Conclusion

Our data reported here, for the first time, indicates that SDF mitigates dentin caries due to inter-kingdom, polymicrobial plaque biofilm growth whilst arresting mineral loss and lesional growth. There was no difference in the caries-arresting efficacy of Saforide, Topamine, and a Thai T-SDF product, all containing 38% SDF.

## Supporting information

S1 Fig Supplementary data: Flowchart of the study. (TIF)
