# Supplementary figures and images for: Efficacy of silver diamine fluoride (SDF) in arresting dentin caries against inter-kingdom biofilms of Streptococcus mutans and Candida albicans
Source: PLoS One. 2024 Sep 30;19(9):e0308656. doi: 10.1371/journal.pone.0308656 (PMC11441700; doi:10.1371/journal.pone.0308656)

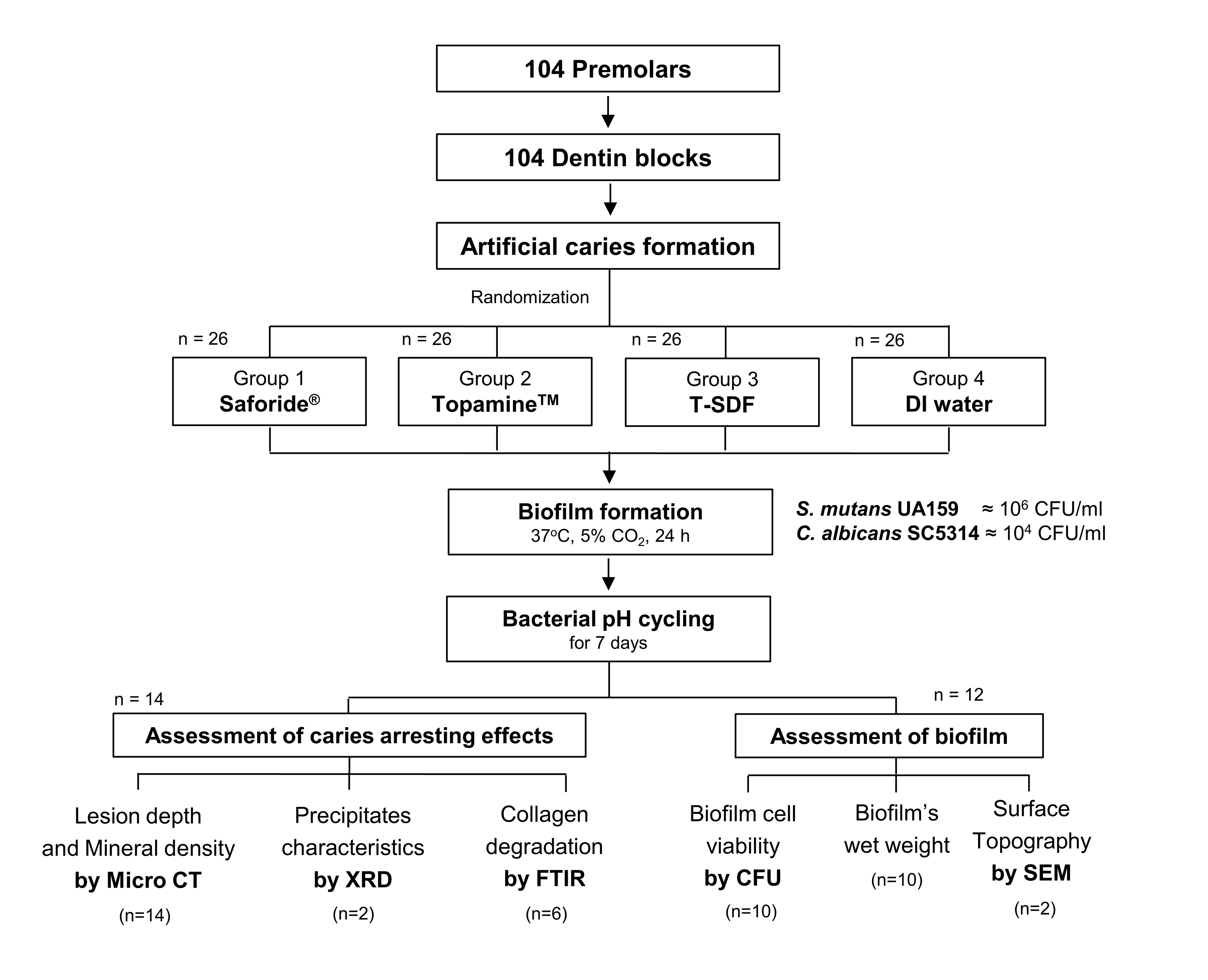

Supplement: S1 Fig — (TIF) [file pone.0308656.s001.tif]
